# Agile Co-Creation for Robots and Aging (ACCRA) Project: new technological solutions for older people

**DOI:** 10.1007/s41999-018-0106-7

**Published:** 2018-09-06

**Authors:** Grazia D’Onofrio, Laura Fiorini, Marleen de Mul, Isabelle Fabbricotti, Yasuo Okabe, Hiroshi Hoshino, Raffaele Limosani, Alessandra Vitanza, Francesca Greco, Francesco Giuliani, Denis Guiot, Eloïse Senges, Antonio Kung, Filippo Cavallo, Daniele Sancarlo, Antonio Greco

**Affiliations:** 1 Complex Unit of Geriatrics, Department of Medical Sciences, IRCCS “Casa Sollievo della Sofferenza”, Viale Cappuccini 1, 71013 San Giovanni Rotondo, Foggia Italy; 2 The BioRobotics Institute, Scuola Superiore Sant’Anna, Pontedera, Italy; 3 Erasmus School of Health Policy and Management, Erasmus University Rotterdam, Rotterdam, The Netherlands; 4 Kyoto University, Kyoto, Japan; 5 Connectdot Ltd, Kyoto, Japan; 6 ICT, Innovation and Research Unit, IRCCS “Casa Sollievo della Sofferenza”, San Giovanni Rotondo, Foggia Italy; 7 Centre de recherche DRM-Ermes, PSL, Université Paris-Dauphine, Paris, France; 8 TRIALOG, Paris, France

**Keywords:** Social robot, Elderly, Qualitative research, Needs analysis, Co-creation

## Abstract

**Introduction:**

Worldwide population is getting older. The older persons want to stay independent and wish to increase their engagement in social activities to tackle loneliness, depression, and isolation. Starting from these assumptions, we developed the ACCRA project (Agile Co-Creation for Robots and Aging) with the aim to enable the development of advanced ICT Robotics-based solutions for extending active and healthy aging in daily life by defining, developing and demonstrating an agile co-creation development process.

**Methods:**

ACCRA robotics solutions will be designed and developed to be tested in three different domains: mobility, daily life, socialization support in four countries (i.e., France, Netherlands, Italy, and Japan). The proposed approach identifies four different phases: (1) needs analysis, (2) agile co-creation, (3) experimentation, and (4) sustainability analysis. Currently, the first two phases were almost completed. For the needs phase, we have used the following recruitment criteria: (1) for mobility: age ≥ 60 years, the and presence of mobility issues assessed by Older Mobility Scale (EMS) with a score > 13; (2) for daily life: age ≥ 60 years, and the presence of difficulties engaging in housework assessed by Autonomie Gérontologie Groupes Iso-Ressources (AGGIR) with a GIR score ≥ 4; (3) for socialization support: age ≥ 60 years, and the absence or mild level of cognitive impairment assessed by Mini Mental State Examination (MMSE) with a score ≥ 24.

**Results:**

The needs analysis and first co-creation sessions focus attention on the experience of older in the four countries. Preliminary results showed how, in all the pilot sites, many expectations were raised from older, formal and informal caregivers about the application of the technology into their life. Minor concerns existed about privacy, real efficacy and modularity in a real-world environment. Overall, a good attitude was recorded towards the use of technologies to support life and promote independent living. Moreover, the older engaged in our studies showed a great interest to be actively involved in the developing phase of something built based on their needs.

**Conclusions:**

The availability of new solutions to increase independence and quality of life in a sustainable manner appears to be mandatory in the actual society considering the actual socio-economic situation over the industrial countries.

## Introduction

The worldwide older population is expected to increase [1], and the sustainability of the entire health care system will be a crucial argument for discussion in the near future.

Latest tendencies focus the design of acceptable and usable products on specific stakeholders needs. In general, older persons want to stay independent and in close contact with their families. They do not want to be considered as a burden for society and wish to increase their engagement in social activities to tackle loneliness, depression, and isolation.

Particularly, adherence to the therapies could have a crucial impact on subjects with chronic diseases and often, the consumption of multiple drugs per day and the use of different devices, make the process very difficult.

Potentially new technological approaches, also based on robotics, might be an effective answer to these growing needs and demands. Actually, some evidence begin to arise regarding their usefulness [2, 3]. Several research experiences outlined remarkable reactions of interest from the older involved in experimentations with assistive robots, especially in terms of impact in support activities, cognitive stimulation, inclusion and reduction of loneliness [4–6].

Social robotics rises to the challenge of designing robots able to perceive the needs and feelings of users with adaptation intentions. It is plausible that, under this perspective, if robots were able to show these skills, they would be accepted as social companions in a more easy way. With these assumptions, the Agile Co-Creation for Robots and Aging project (ACCRA) was proposed in reply to the EU H2020-PHC-19-2014 call. It involves four countries (i.e., France, Netherlands, Italy, and Japan) and it is funded by a European Union–Japan cooperation program with the aim to enable the development of advanced ICT Robotics-based solutions for extending active and healthy aging through an agile co-creation development process. The peculiar aim is to define a specific standard in the development methodology to improve and maintain the level of autonomy, while simultaneously promoting the maintenance of social ties of the older people with loss of autonomy.

## Methods

### ACCRA methodology

The ACCRA methodology to design and evaluate technology in the context of aging well brings together expertise from robotics, software development, marketing, health services research, and health economics, which is the peculiarity of the proposed approach.

It can be considered an innovative approach, which aims to build a new framework on how to conduct these kinds of research experiences, taking into account and identifying the needs of the older people experiencing loss of autonomy, positioning them and their caregivers in the heart of the creation process. Specifically, the methodology shall use a strategy to co-create robotic solutions that meet the users’ needs, to evaluate their daily use and to measure the sustainability of the ACCRA solutions.

The proposed approach identifies four different phases (i.e., steps):

1. *Needs analysis*: identifying needs and investigating the context for each application use.
2. *Agile co*-*creation*: developing the robotics solutions in conjunction with the older, the informal and formal caregivers, using agile design and development tools.
3. *Experimentation*: wide-scale testing of the robotics solutions in a real context.
4. *Sustainability analysis*: defining the potential market interest and assessing the impact of robotics solutions built for the health system.

### Definition of scenarios

The proposal is focused on three different scenarios: (1) mobility, (2) daily life and (3) socialization support. During the project, some applications and services will be developed using two different robots: (1) ASTRO robot [7] to provide both physical and non-physical support and, (2) Buddy to improve socialization and daily life support.

The *mobility application* (involved pilot sites: Netherlands and Italy) essentially supports and coaches the older in the activity of walking; in particular, the ASTRO robot should help users to stand up and walk (i.e., physically as a coach) contributing to maintain and improve the walking capacity. These applications are focused on people with high risk of falls or undergoing post-stroke rehabilitation.

The *daily life application* (involved pilot sites: France and Netherlands) tries to promote well aging in activities of daily life. The companion robot (i.e., Buddy robot) helps users on daily-life activities at promoting independence and well-being (e.g., daily companionship, protection and safety, improving social link, wellness, entertainment, etc.) and for detecting potential risks of autonomy reduction.

Finally, the *socialization application* (involved pilot sites: Italy and Japan) enables diversified communication channels (e.g., remote video conferences, sharing photos, playing games, etc.) to induce conversation rehabilitation. This last is focused on creating interaction schemes with older people in a process similar to physical rehabilitation, where medical professionals assign rehabilitative exercises to the patients. Socializing and keeping conversations (also through a virtual community), maintaining a quality link with the caregivers despite the distance, conversation rehabilitation to engage people in conversational activities, besides the use of companion robots with verbal interaction capabilities are some of the goals of the project to promote a good level of independent living. Simultaneously, those activities may induce both entertainment and challenging interactions based on the patient intellectual curiosity (i.e., preferences and psychological profile) [8].

## Needs analysis and agile co-creation

The purpose of the needs analysis is the identification of desires and behaviors of the older in loss of autonomy based on a qualitative approach consisting of in-depth interviews. Indeed, their needs are essentially related to the physical and physiological disorders due to functional decline and chronic diseases. By placing all involved stakeholder at the center of the innovation process, the aim of the project is to design robotic solutions and services that effectively meet needs, expectations and habits of older people with loss of autonomy and/or social isolation and their caregivers. The participation of all involved stakeholders helps to improve the robotic solution and services by proposing concrete optimization solutions, perceived as effective by the older people, family and professional caregivers and, finally, robotics technicians.

This approach is strategic and the introduction of working groups, named *co*-*creation groups*, which work together during common co-creation sessions, formalizes this social innovation [9, 10] in the ACCRA project. Indeed, researchers in social innovation show how involving the diversity of actors in the innovation process with an active participation of users is an essential feature in the creation and implementation of new solutions. This mechanism tries to provide a more complete representation of the problems, causes and possible solutions to better deal with the identification of individual issues. This finally encourages the implementation of the desired solutions [11].

The study of needs and the co-creation meetings were performed by the WVO Zorg in Vlissingen (Netherlands), the Complex Unit of Geriatrics of IRCCS “Casa Sollievo della Sofferenza” in San Giovanni Rotondo (Italy), CCAS (Centre Communal d’Action Sociale) in Antibes (France), at Fukujuen (social welfare corporation, located in Aichi Pref. Japan) and in Kyoto lighthouse Suzaku dorm (older general welfare center, located Kyoto Pref. Japan).

For what concerns the co-creation sessions, the participants (i.e., older people and formal/informal caregivers) were recruited by the different care organizations. Healthcare professionals explained the goals of the project to the participants, also with letters and brochures about the project.

The recruitment inclusion criteria for mobility were: (1) age ≥ 60 years, and (2) the presence of mobility issues assessed by Older Mobility Scale (EMS) with a score > 13 [12]. The recruitment inclusion criteria for daily life were: (1) age ≥ 60 years and (2) the presence of difficulties engaging in housework assessed by Autonomie Gérontologie Groupes Iso-Ressources (AGGIR) with a GIR score ≥ 4 [13]. The recruitment inclusion criteria for socialization support were: (1) age ≥ 60 years, and (2) the absence or mild level of cognitive impairment assessed by Mini Mental State Examination (MMSE) with a score ≥ 24 [14].

The informal caregivers were recruited according to the relationship with the subjects involved in the study. The formal caregivers were recruited on the basis of their older caring experience.

Each session in general took several hours and was recorded in its entirety.

The approval of the study for experiments using human subjects was obtained from the local Ethics Committees on human experimentation. All participants were required to sign three consent forms before participation: consent for participation to the research study, consent for sharing the audio–video-recorded material and data with other consortium members, and a consent form for using the audio–video-recorded material and data for public purposes (publications, dissemination goals).

The sessions were held in native languages. All the observations and annotations of the participants’ ideas regarding the project, inputs for robot improvements and any criticism were recorded and organized.

## Results

The characteristics of all recruited participants are summarized in Tables 1, 2 and 3 according to the different scenarios.

**Table 1:** Mobility application—recruited participants

Country	Activity	Elderly	Informal caregiver	Formal caregiver	Total
Italy	Needs interview	10	15	15	40
	1° co-creation	6	3	6	15
Netherlands	Needs interview	10	–	4	14
	1° co-creation	4	2	5	11
	Total	30	20	30	80

**Table 2:** Daily-life application—recruited participants

Country	Activity	Elderly	Informal caregiver	Formal caregiver	Total
France	Needs interview	10	1	10	21
	1° co-creation	8	2	2	12
Netherlands	Needs interview	10	3	6	19
	1° co-creation	5	1	5	11
	Total	33	7	23	63

**Table 3:** Socialization support application—recruited participants

Country	Activity	Elderly	Informal caregiver	Formal caregiver	Total
Italy	Needs interview	10	15	15	40
	1° co-creation	7	2	1	10
Japan	Needs interview	7	3	3	13
	1° co-creation	3	–	6	9
	Total	27	20	25	72

### Needs analysis

The needs analysis objective was to prioritize the needs and robot services of interest for 3 groups of respondents (older, formal caregivers and informal caregivers).

The results achieved by the need analysis are described according to different ACCRA scenarios, as shown below.

1. Mobility: the results revealed four categories of needs from the perspective of the older persons: instrumental needs, rehabilitation needs, personal safety and activities of indoor daily life. Complementary, three categories of caregivers needs were also distinguished: instrumental needs, rehabilitation monitoring needs and check-up needs. The highest percentage of participants, both older persons and caregivers, showed a positive expectation towards service robotics. Additionally, from the robotics point of view, the robot abilities that should be developed in the next years will deal with motion, interaction, manipulation, decision support and perception abilities.
2. Daily life: in the Netherlands, the needs among the older were classified into three groups: daily life needs, technical and miscellaneous needs, and physical mobility needs.

In France, nine categories of needs have been proposed, by order of priority: needs for companionship, safety needs, communication needs, well-being needs, entertainment needs, meal needs, forgetfulness and loss, buying needs, and dressing-up needs.

The general acceptance level and perception of the robot were decent among the older and caregivers samples. There was interest in the robots and the participants believe that the robot can be useful. Particularly, the safety and reminding services have been appreciated. In addition, the older people believed that the robot could be a nice companion in their daily life.

3. Socialization support: the common needs were categorized into three groups: communication; emotion detection and safety. The Italian older participants have also reported as a priority need the cognitive stimulation, whereas, the Japanese participants have reported the travel, fashion/golf. In addition Italian caregivers had expressed other needs: from the formal caregivers’ perspective, the robot could provide monitoring the patients, whereas from the informal caregivers’ perspective, the robot could offer reminders to take medicines and eat/drink at specific times of the day and support in simple meal preparation, improving, and optimizing the time care. The general robot acceptance level was good and the perception is positive among the participants in the pilot sites.

### First co-creation

Starting from the evaluation of the needs through questionnaires, the first co-creation sessions focused the attention on the experiences of the older in the four countries. Preliminary results showed how, in all the pilot sites, many expectations were raised from the older, formal and informal caregivers about the application of the technology into their life. Minor concerns exist about privacy and real efficacy and modularity in a real-world environment. Overall, a good attitude towards the use of technologies to live an independent life has been recorded. Moreover, the older engaged in our studies showed a great interest to be involved actively in the development phase of something built based on their needs.

During the meetings, the participants were asked for their initial impression at first glance about generic topics, for example, on the ergonomics and the general qualities of robots.

The first co-creation sessions have shown some mixed feelings about robots. Even if it was not asked explicitly, from impressions, it arises that all participants believe that the use of robots is useful to support older and their caregivers. Sometimes, these expectations are beyond the actual capabilities and the aims of the robots and some suggested services would require more higher-level capabilities.

In particular, the specific results were shown in the following for the different scenarios:

1. Mobility: in Dutch and Italian pilot sites, 4 robot services were explored by the participants (walking and guidance, exercise, safety and reminders and monitoring). For the first co-creation session, there were some mixed feelings about ASTRO. It was not asked explicitly, but the participants do believe that the robot could be useful to support the older and the caregivers. However, they had larger expectations for the robot. However, these expectations are beyond the actual capabilities and the aims of the robot. Some services would require the robot to have hands, e.g., handing over medications or performing injections.
2. Daily life: in French pilot site, the tested services are company, protection/security (falls, medication and appointment reminders, good practice reminders), communication (telephone and video calls, reception and sending photos, videos and drawings), and collective animations. The completeness of the offer was a strong vector of positive evaluation. Very high demand was for voice commands (without this, low added value compared to the services available on the internet and on tablet/phone). This is all the more important since the robot was considered too low for tactile controls (difficulty for older people to bend over) and the screen is too small to ensure good visibility. At the Dutch pilot site, the tested services were divided into 4 themes, namely companion robot, protection/security, online communication, and entertainment. The participants were more interested in what the robot could do instead of what it should be able to do to fulfill their needs.
3. Socialization support: in Italian pilot site, seven robot services were explored by the participants (Communication assistance, Protection-Assistance, Protection-Prevention, Protection-Reminder, Entertainment, Collective animation, Companionship). In Japanese pilot site, three robot services were explored (Travel and Fashion/Golf support needs, Promote conversation, Emotion detection). All themes were viewed as important and therefore for the next co-creation session, improvements should be made in each of the themes. Overall, all services were viewed as useful, but optimizations are required to improve the perception of the robot’s performance. For many of the participants, the added value was still difficult to determine.

## Conclusion

In the present manuscript, using a small sample of older people, formal and informal caregivers, the focus is mainly on the ACCRA methodology developed to design and evaluate technology in the context of aging well. The main goal of the ACCRA methodology is to detect and improve the perception, acceptance and usability of the robot by older people.

Clearly, it is too early to draw outcomes and conclusions that could be usable in clinical practice.

The capability of technology to improve the lives of older subjects appears a feasible objective to reach as soon as possible, considering the demographic shift, the economic constraint, and societal changing. The era of artificial intelligence (AI) exploited for socially useful tasks is coming, since these needs have led to the development of an emerging interest in the use of Robotics and AI in Healthcare. Working in this direction, many are the questions to be addressed, like privacy, human–robot interaction, robot acceptability, economic implications, and security. These are not to be considered as barriers but as interesting challenges in which all key players are called to participate to promote an ethically sustainable scientific progress. The use of robots could be considered from the point of view of care professionals as an interesting opportunity to save more time to devote to patient care. This project has also the added value of defining a standardized methodological approach for the development of new technologies that could improve their faster implementation and industrialization.

Moreover, including cognitive support in the design of technological products for domestic assistive services can be more useful in terms of independent living both at home and in the community. Clearly, it is difficult to imagine how society could change after the massive introduction of humanoid robots capable to perform any work but, in the project we described in this paper, we focused our attention on robotic tools that, more than representing a threat, might improve the lives of many subjects affected by disabilities.
